# Mental Health Problems in Adolescence and the Interpretation of Unambiguous Threat

**DOI:** 10.1371/journal.pone.0127167

**Published:** 2015-06-03

**Authors:** Julie D. Henry, Ernestina Moses, Julieta Castellini, James Scott

**Affiliations:** 1 School of Psychology, University of Queensland, St Lucia, QLD, Australia; 2 School of Medicine, The University of Queensland Centre for Clinical Research, St Lucia, QLD, Australia; 3 Australia Metro North Mental Health, Royal Brisbane and Women’s Hospital, Herston, Queensland, Australia; University of Granada, SPAIN

## Abstract

Aberrant threat perception has been linked to paranoia, anxiety and other mental health problems, and is widely considered to be a core, transdiagnostic feature of psychopathology. However, to date there has been only limited investigation of whether mental health problems are associated with a biased interpretation of stimuli that have explicit (as opposed to ambiguous) connotations of threat. In the present study, 41 adolescents diagnosed with a mental illness and 45 demographically matched controls were asked to provide danger ratings of stimuli normatively rated as being either low or high in potential threat. All participants were also asked to complete background measures of cognitive function, mental health and wellbeing. The results indicated that the two groups did not differ in their capacity to discriminate between low and high threat stimuli, nor did they differ in the absolute level of threat that they attributed to these stimuli. However, for the control group, the overall level of threat perceived in facial stimuli was correlated with two important indices of mental health (depression and anxiety). No associations emerged in the clinical group. These data are discussed in relation to their potential implications for the role of aberrant threat perception in transdiagnostic models of mental health.

## Introduction

Threat perception refers to how an individual detects, attends, interprets and responds to environmental stimuli that may pose risk or danger [1]. Threat perception is therefore an evolutionarily important capacity, fundamental to survival and adaptive functioning [2]. Reduced capacity to accurately and rapidly detect threat in one’s immediate environment has direct implications for personal safety and wellbeing. In a social context, it is vital to be sensitive to the information conveyed in facial expressions to assess whether a specific individual is a source of potential harm or danger. In a non-social context, it is critical to be able to quickly and accurately infer objective risk from fearful and threatening scenes such as attacks, explosions and mutilations. Conversely, a tendency to overestimate threat in either of these contexts could result in an inefficient state of constant anxiety and hypervigilance [3].

To date, literature on threat perception and mental health has involved two major areas of inquiry; *attention* to threat-relevant stimuli and *interpretation* of threat-relevant stimuli. Indeed, in their cognitive model, Hirsch and Mathews [4] argue that habitual biases in attention and interpretation favouring threat content are amongst the most important involuntary (bottom-up) contributing processes to pathological worry. With respect to attentional mechanisms, various disruptions in the presence of threat-relevant stimuli have been observed in both clinical cohorts [2,5] as well as individuals that present with sub-clinical symptoms of psychopathology [6,7]. Such studies have identified differing patterns of attentional disruption, including an attentional bias away from threat (‘avoidance’) and a vigilance-avoidance pattern [8]. However, the most commonly observed finding is an attentional bias towards threat-relevant stimuli (i.e., ‘hypervigilance’), which does not appear to reflect a bias towards negative stimuli more broadly [9].

Based on findings such as these, it has been proposed that an attentional bias towards threat may reflect a core, transdiagnostic factor, linked to both initial onset and subsequent maintenance of mental illness. Thus, it has been argued that an individual who selectively attends to threatening stimuli will live in constant hypervigilance, which leads to maladaptive cognitive, attentional and behavioural patterns (e.g. avoidance, catastrophising, social withdrawal) that each contribute to the development of psychopathology [10]. Any ongoing tendency to selectively attend to the most threatening aspects of one’s environment will then continually reinforce this perception of ever-present danger [1,11,12,13].

As noted, there have also been a number of studies focused on how the *interpretation* of threat-relevant stimuli is affected by psychopathology. Threat interpretation refers to how readily an individual attributes threatening intentions or characteristics to environmental stimuli [14], and cognitive models of anxiety propose that the tendency to interpret *ambiguous* information as threatening also plays a causal role in the etiology and maintenance of pathological anxiety [15]. In this literature, most studies have focused on better understanding the hostile attribution bias, whereby others’ ambiguous behaviours are interpreted as being motivated by hostility [16]. For instance, in the ‘ambiguous scenario’ methodology, participants are asked to imagine themselves as the protagonist of a scenario (usually social) in which the threat posed is open to interpretation. Participants’ interpretations of such scenarios are used to index how frequently and readily they perceive hostility (i.e. threat) in their environment. Studies in this literature have identified associations between anxiety and depression and a bias towards quick, frequent and intense interpretations of threat [17,18,19,20]. Another approach that has often been taken has been to present participants auditorily with ambiguous homophones (e.g. die versus dye). Individuals with mental health problems have been found to write the threatening spelling of the word more often than controls [13,21].

Taken together, evidence indicates that both initial attention to threat-related cues, as well as interpretation of ambiguous threat-relevant stimuli, are affected by psychopathology, and reflecting the importance attributed to these biases, considerable focus has been placed on the development of clinical interventions in which these biases are targeted directly [22,23]. However, it remains poorly understood whether there are also abnormalities in the interpretation of stimuli for which there are *un*ambiguous threat cues. Indeed, we are aware of only one study to date involving individuals with mental health problems that has assessed whether threat interpretation abnormalities are seen in relation to such stimuli using validated experimental methods [24]. In this study, participants with schizophrenia and non-clinical controls were shown pictures normatively rated as being either high or low in danger level. Half of the pictures depicted situations (e.g. weather, animals, sporting activities), while the other half showed people’s faces. Although both groups were equally able to differentiate between low and high threat stimuli, people with schizophrenia showed a general tendency to perceive situational stimuli as higher in potential danger, with this bias significantly related to level of positive symptomatology.

These data suggest that the negative interpretation bias associated with psychopathology may not be specific to ambiguous stimuli. However, an important consideration is that the participants in Henry et al.’s [24] study were all diagnosed with a severe mental illness (schizophrenia), and had a long history of being unwell (on average 23 years since initial diagnosis). Confounding social factors associated with chronic mental illness such as unemployment, unstable accommodation and social isolation rather than mental illness may therefore have accounted for the negative interpretation bias to unambiguous stimuli. Examining this capacity in adolescents whose illness is of relatively recent onset is therefore needed to gain greater insight into the nature and specificity of threat perception abnormalities associated with mental illness. Consequently, the aim of the present study was to identify whether threat interpretation abnormalities are also evident in a mixed-clinical cohort of adolescents using Henry et al.’s [24] stimuli. Adolescence represents a critical developmental period in which most adult mental health problems first emerge, and there is now considerable literature showing that adolescents with mental health problems are similar to their adult counterparts in showing a tendency to interpret ambiguous information as threatening [23]. However, potentially important differences between adolescents and adults with mental health problems have also been identified, such as in the patterns of neural activation shown when appraising threat [25]. The present study will provide important clarification on whether the abnormalities interpreting threat in *un*ambiguous stimuli reflect a relatively early, potentially transdiagnostic feature of mental illness, or whether they are instead specifically linked to schizophrenia and/or the adverse social experiences of those with chronic mental illness.

## Method

### Ethics statement

Ethics approval was obtained from Royal Brisbane and Women’s Hospital Human Research Ethics Committee (#2012000130). All participants provided written informed consent prior to taking part in the study. Informed consent was also obtained from the next of kin, caretakers or guardians on behalf of the minors enrolled in the study. The consent here however was verbal, not written, as often it was only possible to make contact with next of kin, caretakers or guardians via telephone. Once their consent was obtained, this was recorded in writing by the experimenter. This procedure for obtaining consent was approved by the Ethics Committee.

### Participants

Forty-one clinical participants aged between 14 and 17 years of age were recruited from the Adolescent Inpatient Unit of the Royal Brisbane and Women’s Hospital. During this time, a total of 107 individuals were admitted as inpatients. Potential participants were identified by members of the treating team and were not deemed eligible if they were cognitively impaired (IQ < 70), acutely psychotic or if consent could not be obtained. The reasons for non-participation were as follows: 24 were deemed inappropriate due to illness severity, 33 were inpatients for too short a period to permit assessments to be conducted, for six it was not possible to obtain parental consent and three refused to participate. Clinical information was obtained from the treating team, with diagnoses made by treating child and adolescent psychiatrists.

For approximately a third of the clinical sample (32%) major depressive disorder was the primary diagnosis. Other primary diagnoses included anxiety disorder (19%), anorexia nervosa (17%), psychotic disorder (10%), conversion disorder (7%), and bipolar disorder (2%). The majority (58.5%) of the sample also presented with a comorbid mental illness. The mean duration of illness was 17.6 months (*SD* = 13.40), and 38 participants (93%) were taking regular psychotropic medication, with 20 (49%) taking two or more types. Eighty-one per cent were prescribed an antidepressant, 42% were taking antipsychotic medication and 32% were prescribed a benzodiazepine. On average, clinical participants were tested 14.0 days (*SD* = 21.48) following hospital admission.

Forty-five non-clinical participants were recruited from the general community via a combination of methods, including community advertising and snowballing. Exclusion criteria included a current or previous mental illness, treatment by a counsellor, psychologist or psychiatrist, use of psychotropic medication or cognitive impairment. Participants in the clinical and non-clinical groups did not differ in age (*M* = 15.4, *SD* = 1.02, and *M* = 15.1, *SD* = 1.18, respectively, *t*(84) = 1.26, *p* = .213; years of education (*M* = 10.4, *SD* = 0.99, and *M* = 10.3, *SD* = 1.22, respectively, *t*(84) = 0.52, *p* = .604); or gender (81% vs 82% female, respectively, *χ* ^*2*^(1, *n* = 86) = .043, *p* = .836).

### Materials and Procedure

The following background measures were administered: The Vocabulary subtest of the Wechsler Abbreviated Scale of Intelligence [26], the 30-item Reynolds Adolescent Depression Scale (RADS-2 [27]); αs = .83 and .86 for the clinical and non-clinical groups, respectively, the 49-item *Revised Children’s Manifest Anxiety Scale* (RCMAS-2 [28]); αs = .91 and .86, respectively, and the five-item *Satisfaction with Life Scale* (SWLS [29]); αs = .79 and .86, respectively. All of these measures have been shown to have good reliability and validity, and to be appropriate for use in adolescent cohorts, specifically.

#### Danger Rating Task—Faces

We used the stimuli of Ruffman et al. [30] to examine participants’ ratings of danger in faces. These were 20 black-and-white photographs of people’s faces taken from a larger stimuli set of 100 faces developed by Adolphs, Tranel and Damasio [31]. The initial 100 faces were selected for their good reliability, indicated by control participants’ low variance in each face’s ratings of trustworthiness and approachability. These ratings were supported by the pilot study of Ruffman et al. [30], with healthy adults reporting analogous ratings of the faces in terms of danger. Specifically, the ten faces that were judged most approachable were also judged to be the least dangerous, and the ten faces that were judged least approachable also judged to be the most dangerous. The typical low-danger individual was young, female and smiling, whereas the typical high-danger individual was middle-aged, male and not smiling. The bias linking a smiling female with low danger and an unsmiling male with high danger provide the stimuli with ecological validity. Thus, there is good evidence for the reliability and validity of these stimuli as representative of unambiguously low and high danger faces.

#### Danger Rating Task—Situations

The pictures for the situation task were also black and white photos, and were selected by Ruffman et al. [30] from a larger set of 47 pictures. The pictures included different activities (e.g., rally car driving vs. swimming), animals (e.g., tiger vs. kittens), and environmental conditions (e.g., storm clouds vs. non-storm clouds). Human faces were not present in the photos depicting situations. The ten high danger situations and the ten low danger situations were again selected based on normative judgements of non-clinical volunteers [30]. Used together, these Danger Rating Tasks have proven sensitive to group differences when comparing age groups [30] as well as healthy controls to participants with schizophrenia [24] and dementia [32].

Face and situation photos (169 mm height x 132 mm width) were presented 90 degrees to line of sight as separate tasks, with order of task presentation counterbalanced. Items within each of these two tasks were randomized, and were presented one at a time on a computer monitor. While not all stimuli were identical in size, there were no systematic differences between high and low danger exemplars. Participants progressed through the pictures at their own pace. Participants used a mouse to reveal each new photo and made a written response to rate each face and situation on a scale of 1 (not at all dangerous) to 7 (very dangerous). Participants on average took 10–15 minutes to complete each of the two tasks. All participants were observed continuously as they completed the assessments, and on no occasion did any participant go back and change a response.

## Results

### Background Measures

The first step in analyses was to compare the clinical and control groups on the background measures of cognitive function (WASI) and wellbeing (RCMAS, RADS and SWLS). The results showed that the clinical group performed more poorly on the WASI relative to controls (*M* = 95.0, *SD* = 15.01 vs. *M* = 101.3, *SD* = 13.26, respectively; *t*(84) = 2.07, *p* = .041). The clinical group also reported higher anxiety relative to controls (*M* = 26.1, *SD* = 9.60 vs. *M* = 14.8, *SD* = 7.68, respectively; *t*(83) = 5.98, *p* < .001), greater depression (*M* = 88.4, *SD* = 17.74 vs. *M* = 60.4, *SD* = 14.17, respectively; *t*(84) = 8.11, *p* < .001), and lower life satisfaction (*M* = 11.9, *SD* = 6.05 vs. *M* = 24.3, *SD* = 6.98, respectively; *t*(84) = 8.74, *p* < .001).

### Threat Interpretations

**Fig 1:**
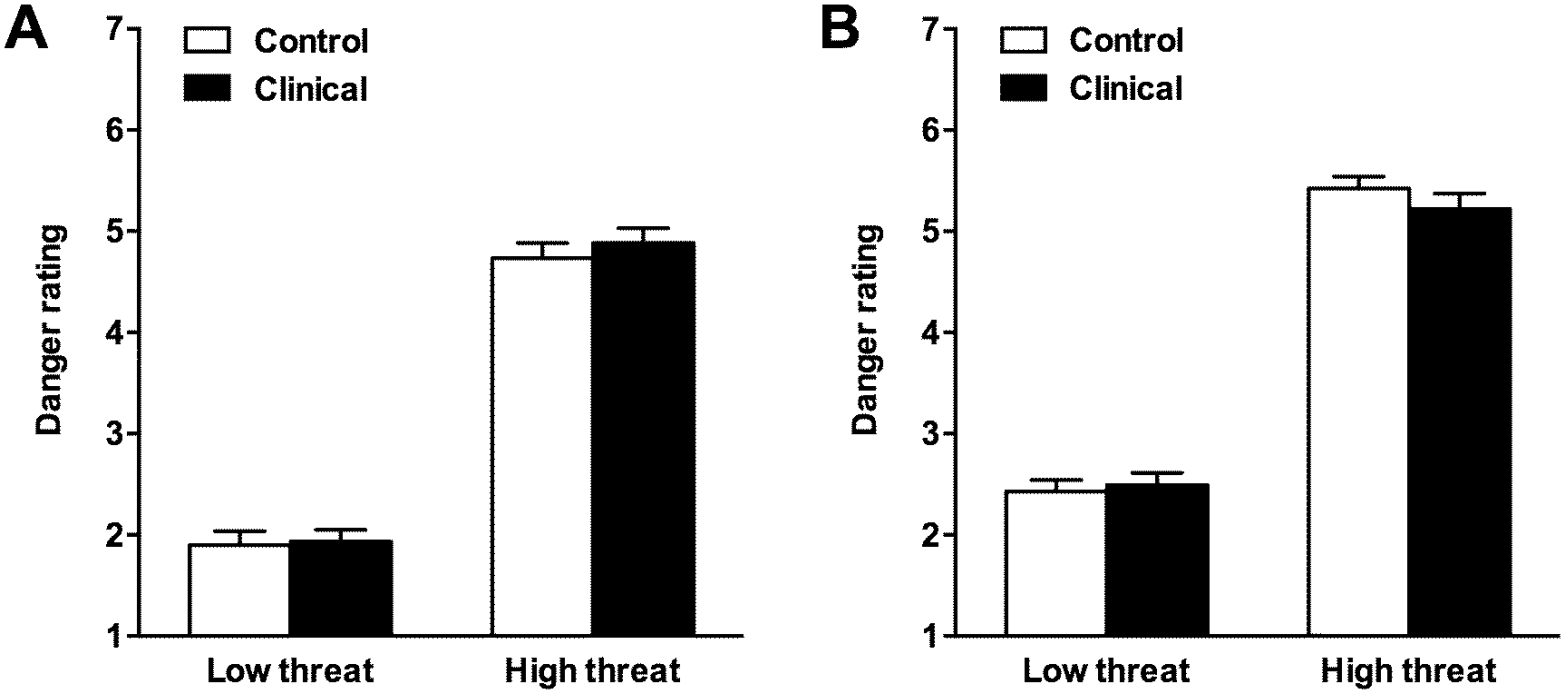
Danger scores (and standard errors) for (A) high- and low-danger faces, and (B) high- and low-danger situations for controls and participants with mental health problems (minimum and maximum scores are 1 and 7, respectively).

Fig 1A and 1B show danger ratings for the clinical and control participants. The Faces data (see Fig 1A) were analyzed with a 2 (group: clinical, control) x 2 (danger level: high, low) analysis of variance (ANOVA), with danger ratings as the dependent variable. These analyses indicated a main effect of danger level, *F*(1,82) = 487.71, *p* < .001, η_p_^2^ = .86, which reflected higher danger ratings on normatively rated high danger than low danger faces. However, there was no main effect of group, *F*(1, 82) = 0.38, *p* = .541, η_p_^2^ < .01, and no interaction between danger level and group, *F*(1, 82) = 0.20, *p* = .66, η_p_^2^ < .01. Thus, as can be seen in Fig 1A, danger ratings for the clinical and control groups were very closely matched, both for high danger faces (*M* = 4.88, *SD* = 0.96, and *M* = 4.73, *SD* = 0.98, respectively), and for low danger faces (*M* = 1.93, *SD* = 0.76, and *M* = 1.90, *SD* = 0.89, respectively).

The Situations data (see Fig 1B) were similarly analyzed with a 2 (group: clinical, control) x 2 (danger level: high, low) ANOVA, with danger ratings as the dependent variable. Again, there was a main effect of danger level, *F*(1,82) = 651.2, *p* < .001, η_p_^2^ = .89, which reflected higher danger ratings on normatively rated high relative to low danger situations. There was also no main effect of group, *F*(1, 82) = 0.27, *p* = .608, η_p_^2^ < .01, and no interaction between danger level and group, *F*(1, 82) = 1.41, *p* = .239, η_p_^2^ = .02. As can be seen in Fig 1B, danger ratings for the clinical and control groups were very closely matched, both for high danger situations (*M* = 5.22, *SD* = 0.98, and *M* = 5.43, *SD* = 0.77, respectively), and for low danger situations (*M* = 2.49, *SD* = 0.76, and *M* = 2.43, *SD* = 0.73, respectively).

### Correlates of Danger Ratings

**Fig 2:**
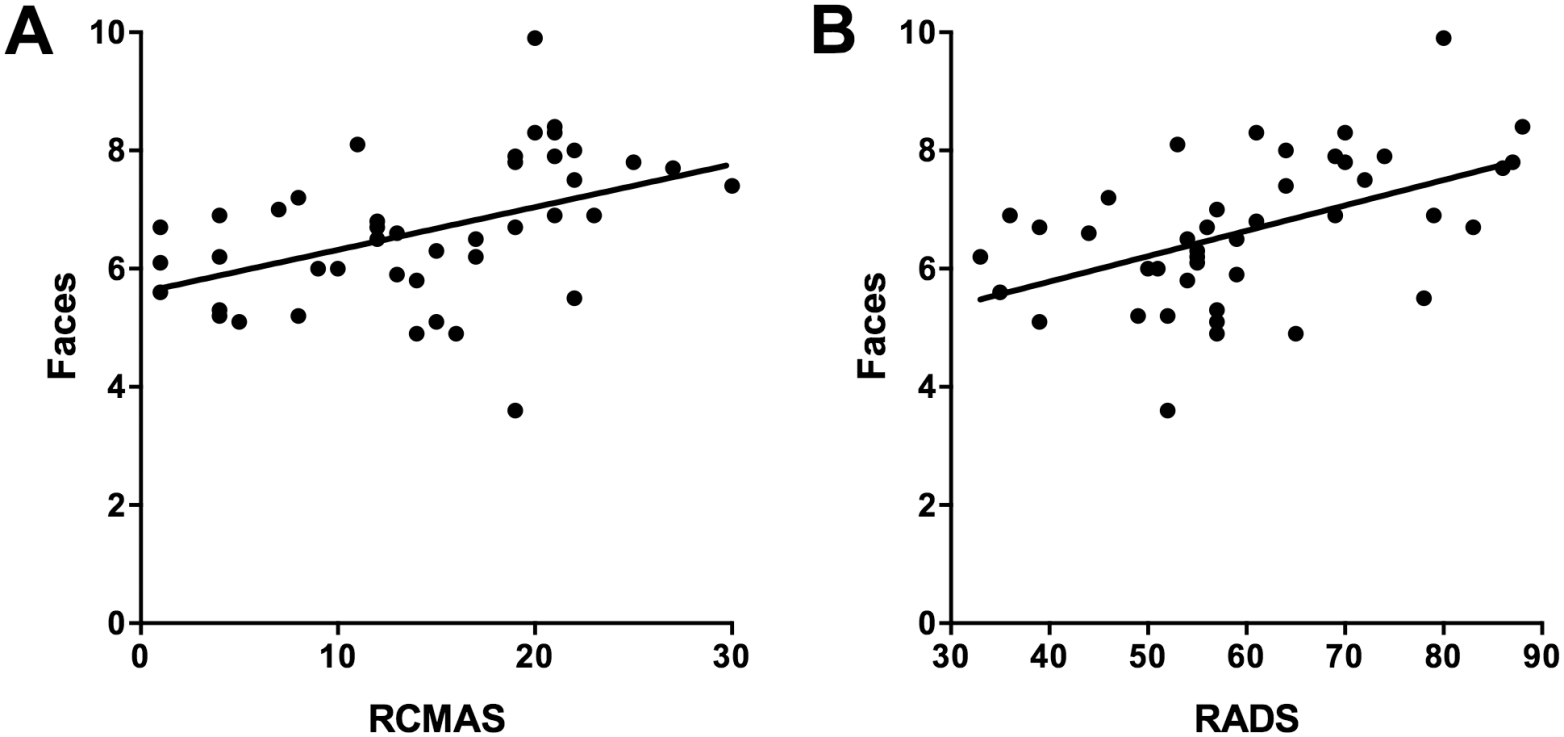
Scatterplots depicting associations between total danger scores for faces and (A) scores on the RCMAS, and (B) scores on the RADS.

Threat interpretation data were then examined in terms of their correlates with cognitive function, mental health and satisfaction with life. Because both groups were equally able to differentiate between high and low threat exemplars, for these analyses we computed a total danger rating score separately for faces and situations. These correlations are reported in Table 1, separately for the two groups. Because a relatively large number of correlations are reported, a Bonferonni correction was applied. To avoid inflating Type II errors of interpretation, the appropriate way to apply this correction is to adjust the alpha level for each comparison not only in relation to the number of comparisons made (i.e. 16), but also in relation to the inter-correlation between the four dependent variables (average intercorrelation between the four dependent variables was .64). This procedure yields a corrected *p* value of .018. Applying this corrected value, in the clinical group, threat ratings were unrelated to all dependent measures. However, in the control group, two significant correlations emerged. Specifically, higher threat ratings to face stimuli were significantly correlated with both measures of psychopathology (RCMAS, *p* = .003; RADS, *p* = .001). According to Cohen’s [33] criteria, these correlations were both moderate to large in magnitude. It can also be seen from the scatterplots depicting these associations in Fig 2A and 2B that there is no evidence that either of these correlations are artefactual.

**Table 1:** Correlates of threat attributions.

Measure	Control (*n* = 43)		Clinical (*n* = 41)	
	Faces	Situations	Faces	Situations
*Cognitive function*				
WASI	-.13	-.21	-.11	-.18
*Psychopathology/ wellbeing*				
RCMAS	.44*	.26	.15	.19
RADS	.50*	.24	.12	-.02
SWLS	-.33	-.04	.02	.18

*Note*, *WASI*: Wechsler’s Abbreviated Scale of Intelligence; *RCMAS*: Revised Children’s Manifest Anxiety Scale; *RADS*: Reynolds Adolescent Depression Scale; *SWLS*: Satisfaction With Life Scale.

*Denotes statistically significant, after applying Bonferroni correction.

## Discussion

The present study shows for the first time that adolescents with mental health problems do not differ from controls in their ability to detect unambiguous threat from either the facial expressions of others or from non-social situations. Thus, no group differences emerged with respect to either overall levels of danger perceived, or in the ability to differentiate normatively judged high- and low-danger faces, or high- and low-danger situations. These data therefore deviate from the only study to date that has assessed this type of threat perception in an adult psychiatric cohort. As noted, Henry et al. [24] found that although individuals with schizophrenia did not differ from controls in their ability to differentiate high- and low-threat faces or situations, they provided higher threat ratings overall to situational stimuli. Specifically, they exhibited a general bias to perceive situational stimuli as being higher in potential danger. The present data indicate that these negative interpretational tendencies are not an early feature of mental illness, but may instead be specifically linked to a diagnosis of schizophrenia and/or the adverse social consequences of a chronic mental disorder. Consistent with the former of these possibilities, Henry et al. [24] identified a significant association between positive symptoms and a negative threat interpretation bias, suggesting a potential specific schizophrenia-related abnormality.

These data also deviate from prior studies in the threat interpretation literature that have investigated responses to ambiguous stimuli. As noted, with few exceptions these studies have shown that psychopathology—both clinical and subclinical—is related to quicker, more frequent and more intense interpretations of threat [13,18,19,20,21,22]. The present results raise the interesting possibility that in adolescence at least, this bias may reflect an ambiguity-dependent effect, and consequently may be neither as pervasive nor problematic as previously thought. Such a possibility is particularly interesting in light of the fact that studies that have identified attentional disruptions have most often used stimuli that is unambiguous with respect to potential threat, as was the case in the present study. The finding of abnormalities in attention—but not interpretation—of unambiguous threat cues therefore indicates that these different components of threat perception may be dissociable.

Given the critical importance of being able to make appropriate interpretations of threatening and non-threatening stimuli in our everyday environment, the finding that mental health problems in adolescence does not affect this capacity is potentially clinically significant. As noted earlier, both hyper-and hypo-vigilance to threat have been linked to negative functional outcomes in everyday life, and indeed the second major finding to emerge in the present study was that higher ratings of threat to the facial stimuli were associated with poorer mental health in the control group. Thus, control participants who reported seeing greater threat in facial stimuli were also those who reported the highest levels of anxiety and depression. The absence of these associations in the clinical group however, perhaps suggests that once a clinically significant level of psychopathology is reached, there is no further tendency to appraise others as more threatening i.e. a ceiling effect is reached. Alternatively, the correlations identified in the control group might reflect associations between threat interpretation and individual differences in personality traits related to anxiety or depression proneness, not state anxiety or depression *per se*. Consistent with such a possibility, Doty et al. [34] showed that sensitivity to detecting *un*ambiguously threatening faces varied parametrically across the healthy population, and was associated with anxiety-related traits, but not situational fluctuations in anxiety. Finally, it is also important to note that the identification of distinct functional correlates for facial and situational threat also perception aligns with other studies that have also shown that there are meaningful differences between the ability to perceive threat from facial relative to non-facial cues [24,30,32].

### Limitations and Future Directions

The present data need to be considered in light of a number of potential limitations. First, a mixed clinical sample was used, making it difficult to generalise to specific disorders. However, this limitation is also an important strength, given that abnormal threat perception is widely regarded as a transdiagnostic factor. As noted previously, the high incidence of comorbidity in clinical samples and an overlap in diagnostic classification, have pushed towards a trend for mental illnesses to be viewed in terms of the commonalities they share [35]. Nevertheless, future research is now needed to cross-validate these findings in other, specific clinical samples.

A second limitation is that it might be argued that the stimuli used to assess threat interpretation were inappropriate for use in adolescent cohorts, having been designed for and piloted solely with adults [24,30,31,32]. However, both groups of adolescents were able to accurately discriminate between low and high threat faces and situations. That is, the danger ratings of both groups yielded the same categorisation of images into low and high threat as has been observed in previous studies. This accurate discrimination between low and high threat pictures indicates that the stimuli were appropriately perceived as representative of the specific threat levels intended. Finally, the study was cross-sectional which prevents any conclusions to be made as to the direction of the association between psychopathology and threat perception.

In addition, while the present results are important in that they provide novel insights into how threat perception is affected in adolescents with mental health problems, future research is now needed in which both attentional and interpretative biases are assessed in a single study, ideally in response to both ambiguous and unambiguous stimuli. Such a design would substantially enhance our understanding of when and why psychopathology might be expected to affect threat perception. It would also be valuable to include measures of perceived control. Regulatory control has been argued to moderate the relationship between threat-related interpretive bias, and its modification in late childhood and adolescence [36,37]. Indeed, in Hirsch and Mathew’s [4] cognitive model of pathological worrying, involuntary (bottom-up) biases in attention and interpretations that favour threat content are argued to interact with voluntary (top-down) processes such as attentional control.

Finally, future studies focused on the interpretation of unambiguous threat should incorporate not only measures of accuracy, but also reaction time. Potentially important differences in response speed might be evident, even in the absence of any differences in explicit ratings of perceived threat. Indeed, in the related attentional bias literature, Van Damme and Crombez [38] showed that an attentional bias to threat in a non-clinical youth sample only emerged when fast reaction times were taken into account. In a related manner, participants in the present study were allowed to progress through the Danger tasks at their own pace to ensure that they had sufficient time to encode and make sense of the stimuli. However, given that in everyday life, interpretations of threat often need to be made very quickly, future research should manipulate the speed of stimuli presentation, and in particular, assess performance when stimuli is presented at a fixed, rapid pace. Together, through a combination of methods such as these, it will be possible to arrive at a fuller, more nuanced understanding of how basic aspects of threat perception and interpretation are affected by the presence of mental health problems in adolescence.
